# AN1284 attenuates steatosis, lipogenesis, and fibrosis in mice with pre-existing non-alcoholic steatohepatitis and directly affects aryl hydrocarbon receptor in a hepatic cell line

**DOI:** 10.3389/fendo.2023.1226808

**Published:** 2023-08-16

**Authors:** Adi S. Yehezkel, Nathalie Abudi, Yuval Nevo, Hadar Benyamini, Sharona Elgavish, Marta Weinstock, Rinat Abramovitch

**Affiliations:** ^1^ The Goldyne Savad Institute of Gene Therapy, Hadassah Medical Center, Faculty of Medicine, Hebrew University of Jerusalem, Jerusalem, Israel; ^2^ The Wohl Institute for Translational Medicine, Hadassah Medical Center, Jerusalem, Israel; ^3^ Info-CORE, Bioinformatics Unit of the I-CORE at the Hebrew University of Jerusalem, Jerusalem, Israel; ^4^ Faculty of Medicine, School of Pharmacy, Institute for Drug Research, Hebrew University, Jerusalem, Israel

**Keywords:** aryl hydrocarbon receptor, nuclear receptors, oxidative stress, retinoid X receptor, RNA-sequencing analysis, fatty acid synthase

## Abstract

Non-alcoholic steatohepatitis (NASH) is an aggressive form of fatty liver disease with hepatic inflammation and fibrosis for which there is currently no drug treatment. This study determined whether an indoline derivative, AN1284, which significantly reduced damage in a model of acute liver disease, can reverse steatosis and fibrosis in mice with pre-existing NASH and explore its mechanism of action. The mouse model of dietary-induced NASH reproduces most of the liver pathology seen in human subjects. This was confirmed by RNA-sequencing analysis. The Western diet, given for 4 months, caused steatosis, inflammation, and liver fibrosis. AN1284 (1 mg or 5 mg/kg/day) was administered for the last 2 months of the diet by micro-osmotic-pumps (mps). Both doses significantly decreased hepatic damage, liver weight, hepatic fat content, triglyceride, serum alanine transaminase, and fibrosis. AN1284 (1 mg/kg/day) given by mps or in the drinking fluid significantly reduced fibrosis produced by carbon tetrachloride injections. In human HUH7 hepatoma cells incubated with palmitic acid, AN1284 (2.1 and 6.3 ng/ml), concentrations compatible with those in the liver of mice treated with AN1284, decreased lipid formation by causing nuclear translocation of the aryl hydrocarbon receptor (AhR). AN1284 downregulated fatty acid synthase (FASN) and sterol regulatory element-binding protein 1c (SREBP-1c) and upregulated Acyl-CoA Oxidase 1 and Cytochrome P450-a1, genes involved in lipid metabolism. In conclusion, chronic treatment with AN1284 (1mg/kg/day) reduced pre-existing steatosis and fibrosis through AhR, which affects several contributors to the development of fatty liver disease. Additional pathways are also influenced by AN1284 treatment.

## 1 Introduction

Non-alcoholic steatohepatitis (NASH) is an aggressive form of non-alcoholic fatty liver disease (NAFLD) with an excess of fatty acids and triglycerides, lobular inflammation, hepatocyte injury, and fibrosis (1), accompanied by insulin resistance and oxidative stress (2). Insulin resistance promotes lipogenesis through an influx from adipose tissue of free fatty acids (FFAs) into the liver. Oxidative stress impairs fatty acid oxidation, compromising the liver’s ability to use, store, and export FFAs as triglycerides (3). This causes apoptosis to hepatocytes through activation of signal-regulating kinase, which upregulates MAP kinases JNK and p38 (4). Cell damage stimulates hepatic stellate cells to produce TGF-β that induces fibrosis by activating myofibroblasts (5). Other cytokines are produced by FFAs (6) through stimulation of Toll-4-like receptors on Kupffer cells and circulating leukocytes (7) and by the bacterial antigen, lipopolysaccharide (LPS). The concentrations of LPS in the circulation and liver of subjects with NASH are higher than those in controls (8).

Most compounds tested in rodent models of NAFLD or NASH (9–13) (among others) were given with the initiation of the high-fat Western diet (WD), and thus, any effect they had is mainly preventive. A few compounds, each with a different mode of action, were able to ameliorate steatosis when given to mice several weeks after commencement of the diet: Firsocostat, an acetyl-CoA carboxylase (ACC) inhibitor, Tropifexor, an agonist of Farnesoid X receptor (FXR), and cinnabarinic acid, an endogenous agonist of the aryl hydrocarbon receptor (AhR) (14, 15). Firsocostat and Tropifexor arrested the development of fibrosis (15). Although all these drugs reduced steatosis in human subjects with NASH, they had no effect on fibrosis (16). Neither did the novel dual proliferator-activated receptor (PPAR) agonist Saroglitazar (17), although it had fewer adverse effects than other PPAR agonists in humans (18). Thus, there is still a need for safe, clinically effective drugs for treating NASH that can also halt development of fibrosis. The pathophysiology of NASH is complex and probably requires activation of multiple targets for more successful treatment against fibrosis (19).

AN1284 [3-(indolin-1-yl)-N-isopropylpropan-1-amine 2HCl] is a novel drug with multiple actions. It inhibited cytotoxicity resulting from oxidative stress and reduced the release of pro-inflammatory cytokines in LPS-activated macrophages (20) by inhibiting phosphorylation of p38 MAPK and nuclear translocation of Activator protein-1 (21). In mice with acute liver injury caused by LPS/D-galactosamine injection, s.c. injection of AN1284 (0.25–0.75 mg/kg) prevented the elevation of TNF-α and plasma alanine transaminase (ALT) and reduced hepatic damage and mortality (21). Chronic treatment of BSK-db/db mice with type 2 diabetes by AN1284 (2.5 and 5 mg/kg/day) by s.c. implanted micro-osmotic-pumps (mps) before disease development prevented renal damage and reduced elevation of plasma ALT and hepatic fat accumulation, while preserving insulin sensitivity and pancreatic β cell mass (22).

The current study examined the effect of AN1284 (1 and 5 mg/kg/day) administered for 2 months by mps, on hepatic steatosis and fibrosis in mice with pre-existing NASH. This was produced by feeding for 4 months on a modified low-trans-fat Western-diet combined with low choline. RNA sequencing analysis (RNA-seq) confirmed that diet replicated several changes in cellular processes seen in humans with NASH. HUH7 human hepatoma cells were used to show that AN1284 decreased conversion of palmitic acid (PA) to lipid at concentrations compatible with those found in the liver in mice and to elucidate its mechanism of action.

## 2 Materials and methods

### 2.1 *In vivo* NASH studies in mice on WD

Experiments were performed according to the guidelines of the Animal Care and Use Committee of the Hebrew University (NIH approval number OPRR-A01-5011). Male C57BL/6JOlaHsd mice, aged 4 weeks for NASH experiments and 6 weeks for the CCl_4_ fibrosis model (Harlan; Ein Kerem, Israel), were housed (five per cage), in a pathogen-free unit under controlled 12-h light/12-h dark cycle and an ambient temperature of 21 ± 1°C and humidity 40%–50%. The cages contained Teklad Sani-chips (ENVIGO) bedding and two 2" small play tunnels for environmental enrichment. Male mice were selected because they develop a more severe form of the disease than females and have lower antioxidant enzymes (23).

The normal diet (ND) consisted of Teklad 2918SC radiated pellets (ENVIGO) containing 13.9% kcal from fat, 62.9% from carbohydrates, and 23.1% from protein. The modified WD (Envigo-Teklad TD.150235) had 50.5% kcal fat [trans-fat (12% of fatty acids) and saturated fat (50% of fatty acids)], 38% carbohydrates and 11.5% protein, 20% sucrose, 10% fructose, and 1.25% cholesterol with reduced choline (900 mg/kg). This diet, modified from the WD described in Farrell et al. (24), caused hepatic steatosis in mice after 1 month (**Figure S1B**) and inflammation and fibrosis within 4 months.

The mice were maintained for 2 months on WD (*n* = 30) or ND (*n* = 15) and weighed twice weekly. Then, under ketamine 100 mg/kg/xylazine 10 mg/kg anesthesia, they were implanted with mps delivering saline, or AN1284 (1 or 5 mg/kg/day)/month (ND *n* = 5/dose) (WD *n* = 10/dose) for the next 2 months (**Figure S1A**). A new pump was implanted under anesthesia in the second month. In a previous study, there were no significant differences in the effects of 2.5 and 5 mg/kg/day of AN1284 on the parameters measured in diabetic mice (22). Therefore, in the current study, we administered 1 and 5 mg/kg/day. At the end of the experiment, blood was collected by cardiac puncture under ketamine/xylazine anesthesia, and the livers were excised, weighed, and prepared for histological, cytological, biochemical, and molecular analyses.

### 2.2 Induction of liver fibrosis by carbon tetrachloride

Mice (*n* = 25) that were fed with ND were injected i.p. with carbon tetrachloride (CCl_4_) (0.5 mg/kg in corn oil) (Sigma), twice weekly for 7 weeks. Four controls were injected with saline (1 ml/kg). Four weeks later, five mice injected with CCl_4_ were sacrificed and the livers were examined to confirm the presence of fibrosis. The remaining eight mice were given saline by s.c. injection and seven others were implanted with mps delivering AN1284 (1 mg/kg/day) for 3 weeks.

While the current study was in progress, we completed an examination of the pharmacokinetics and metabolism of AN1284 in mice. Peak drug concentrations were similar in plasma and liver after s.c. injection, but nearly 50-fold higher in the liver when the compound was given orally (25). This suggested that oral administration should enable AN1284 to reduce hepatic damage. Therefore, AN1284 (1 mg/kg/day) was given to eight mice (four per cage) for 3 weeks via the drinking fluid, 4 weeks after they had developed fibrosis induced by CCl_4_ injections. Ten others received normal drinking fluid. They were weighed once weekly, their fluid intake was measured twice weekly, and the concentration of AN1284 in the fluid was adjusted accordingly. Seven weeks after commencement of the CCl_4_ injections, the mice were processed for histological and biochemical analyses as described below.

### 2.3 Biochemical and histological analyses

The livers were extracted as described in Ref (26). and their triglyceride content was determined using the Cobas C-111 bio-analyzer (Roche, Switzerland), normalized to wet tissue weight. Plasma ALT was measured by Reflotron chemical blood analyzer (Roche Diagnostics, Mannheim, Germany). Frozen liver was placed in an embedding medium and used for the measurement of hepatic fat content by Oil Red O (ORO) staining. The rest of the liver was fixed for 24 h in 4% formaldehyde solution (Bio-Heart Ltd., Jerusalem, Israel), induced in 70% ethanol and embedded in paraffin, cut into 5-µm slices, and stained with hematoxylin and eosin (H&E) for general damage. Fibrosis was assessed with Sirius Red (SR) (Sigma, 365548), collagen 4 (Col4) (Abcam, ab236640), and immunohistochemical staining with primary antibodies against α-SMA (Sigma, A2547). Antibodies against Ly6B (Bio-Rad, MCA771) were used for neutrophils and natural killer cells, F4/80 for macrophages (Bio-Rad, MCA497), CD3 for T cells (Bio-Rad, MCA1477), CD45R for B cells (Santa Cruz, sc-19597), CD36 (Abcam, ab133625), and iNOS (Abcam, ab3523). Histopathological analysis was performed by a light microscope using the program Cellsens Entry (Olympus, Japan). Macrophages, ORO, α-SMA, and SR were quantified in 12 random images at ×40 magnification. Using the ImageJ software, the colored area was calculated, normalized, and expressed as a percentage of the whole picture.

### 2.4 Quantitative polymerase chain reaction

RNA was extracted from snap-frozen liver tissues (miRNeasy Micro Kit, Qiagen), from six samples/group. Its quantity and integrity were checked (Nanodrop, spectrophotometer) and reverse-transcribed into complementary DNA (qScript cDNA Synthesis Kit, QuantaBio). Genes were determined by PCR with an SYBR Green Kit and (QuantaBio) on the CFX384 Touch Real-Time PCR Detection System (Bio-Rad). The relative expression of target genes was normalized by hydroxyl methyl bilane synthase expression as an internal control. The primer sequences used are listed in **Table 1**.

**Table 1:** A. Mouse primer sequences used for qPCR.

Gene	5’ primer	3’ primer
HMBS	ACTATTGGAGCCATCTGCAAAC	CTCTCCTCAGAGAGCTGGTTC
TNF-α	GAAAAGCAAGCAGCCAACCA	CGGATCATGCTTTCTGTGCTC
IL-10	GGTTGCCAAGCCTTATCGGA	ACCTGCTCCACTGCCTTGCT
CCL2 (MCP-1)	AAGCCAGCTCTCTCTTCCTCCA	GCGTTAACTGCATCTGGCTGA
FASN	CCCCTCTGTTAATTGGCTCC	TTGTGGAAGTGCAGGTTAGG
PLG	ACAGGCACAGCATCTTCACC	CATCTGGGTTTCGGCAGTAGTTC
IL-6	ATACCACTCCCAACAGACCTGTCT	CAGAATTGCCATTGCACAACTC
TGF-β1	ACCAACTATTGCTTCAGCTTACGCTCCAC	GATCCACTTCCAACCCAGGTC

**Table 1:** B. Human primer sequences used for qPCR.

Gene	5’ primer	3’ primer
HPRT	GGACAGGACTGAACGTCTTGC	CAACACTTCGTGGGGTCCTT
FASN	CAAGCTGAAGGACCTGTCTAG	CGGAGTGAATCTGGGTTGATG
ACOX1	ACTCGCAGCCAGCGTTAT	AGGGTCAGCGATGCCAAAC
CYP1a1	ACATGCTGACCCTGGGAAAG	GGTGTGGAGCCAATTCGGAT
SREBP-1C	CTACCGCTCCTCCATCAATG	CTTGAGTTTCTGGTTGCTGTG

### 2.5 RNA sequencing analysis

For RNA-Seq analysis, an Illumina Hi-seq sequencer was used to measure the differences in global gene expression between the experimental groups. Each sample generated approximately 70 × 10^6^ reads at the length of 86 bases. Differential expression data of the whole transcriptome was subjected to Gene Set Enrichment Analysis (GSEA) with the corresponding human ortholog gene symbols. GSEA uses all differential expression data (cutoff independent) to determine whether a priori-defined set of genes show statistically significant, concordant differences between two biological states. The hallmark gene set collection from MSigDB (molecular signature database) was used for the analysis. For each comparison, all statistically significant, differentially expressed genes were subjected to pathway enrichment analysis using QIAGEN’s ingenuity pathway analysis (IPA, QIAGEN Redwood City, www.qiagen.com/ingenuity), GeneAnalytics and EnrichR, and functions/diseases enrichment analysis by IPA.

### 2.6 *In vitro* studies

HUH7 human hepatoma cells were incubated for 24 h in medium containing BSA. To see whether AN1284 can reduce steatosis from an FFA by a direct action on liver cells, PA was added together with different concentrations of AN1284 for 24 h. Lipid content was quantified by ORO staining. Since RNA-seq analysis suggested that AN1284 could act via the aryl hydrocarbon receptor (AhR), we measured the effect of AN1284 on the nuclear translocation of AhR, by immunofluorescence intensity, 15 min after its addition to the cells. We used a specific antibody (Abcam, ab190797), analyzed its intensity with ImageJ, and normalized it to the control group. RT-qPCR was used to measure the target genes of AhR after 24 h: fatty acid synthase (FASN), SREBP-1c, acyl-CoA oxidase 1 (ACOX1), and cytochrome P450-1 (CYP1a1). siRNA for human AhR from TriFECTa Kit DsiRNA Duplex purchased from ITD was used to silence AhR. The reverse transfection of these siRNAs onto HUH7 cells was performed by means of TransIT-X2 Dynamic Delivery System (MC-MIR-6000, Mirus) according to the manufacturer’s instructions. To examine the effect of siRNA on AhR expression, total protein was extracted from cells 48–72 h after transfection, and AhR protein levels were measured using Western blot (WB) with primary AhR antibody (Abcam, ab190797). The siRNA-transfected cells were treated with AN1284 and analyzed.

#### 2.6.1 Protein extraction and Western blotting

Total protein extract was obtained by using Radioimmuno Precipitation Assay lysis buffer for five samples/group. Cell lysates containing 50 μg of total protein were then added to SDS–PAGE gels and transferred to Nitrocellulose membranes (Bio-Rad, 1704158). Membranes were blocked in 1% non-fat milk and incubated overnight at 4°C with primary antibodies, RXRα (Abcam, ab125001), and mouse anti-β-actin (MP Biomedicals, 691001). The signals were developed with an enhanced chemiluminescence solution (Bio-Rad, 1705060) and visualized on a Bio-Rad bioluminescence device. Band intensities were quantified using ImageJ and normalized to actin.

### 2.7 Measurement of hepatic levels of AN1284 and its indole metabolite AN1422

Liver samples were homogenized (100 mg/ml) in phosphate buffered saline. Twenty microliters of internal standard (rivastigmine 750 ng/ml) and 20 µl of ultra-pure water were added. AN1284 and its oxidized metabolite, AN1422, were extracted and measured as described in Weitman et al. (25).

### 2.8 Statistical analysis

Studies were designed to generate groups of equal size whenever possible, and any variation in group size within an experiment was due to unexpected loss of an animal or sample for measurement. All statistical analyses were performed using GraphPad Prism 9.50 (GraphPad Software Inc., San Diego, CA, USA). Data were compared by the Kruskal–Wallis non-parametric method, followed by the Mann–Whitney *post-hoc* test if *F* achieved *P* < 0.05. Body weight changes over time were compared by a two-way repeated measures ANOVA using SPSS version 28. Data are expressed as the mean ± SD. A *p*-value of <0.05 was considered to be significant.

## 3 Results

### 3.1 Liver concentrations of AN1284 and its oxidized metabolite

There were no significant differences in the hepatic concentrations of AN1284 after administration of 1 or 5 mg/kg/day (37.9 ± 9.7 and 51.4 ± 12.0 ng/g), respectively, but those of the indole metabolite, AN1422, were significantly higher after the 5 mg/kg/day dose (3.4 ± 1.1 vs. 9.4 ± 4.9 mg/kg).

### 3.2 AN1284 attenuates liver steatosis

**Figure 1:**
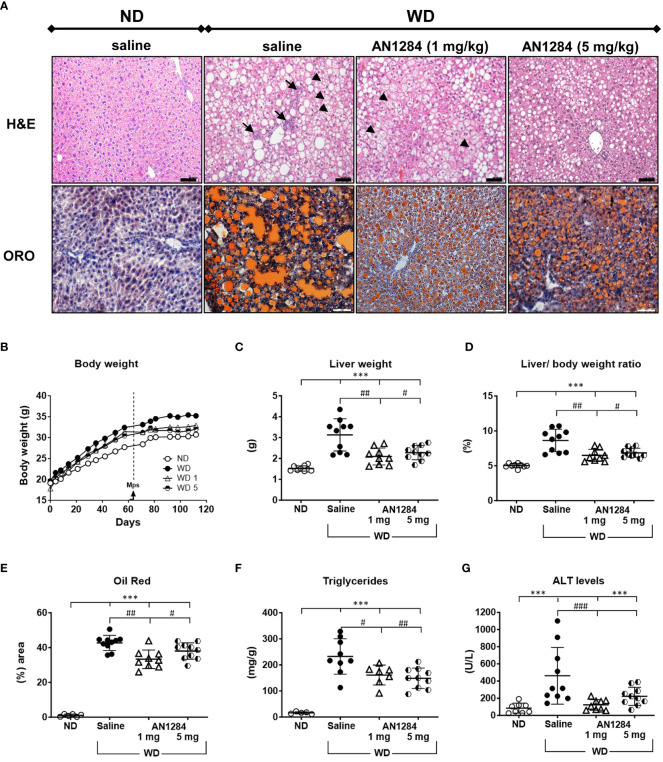
AN1284 reduces cell ballooning, inflammation, and hepatic fat accumulation in mice on a modified Western diet (WD). **(A)** WD causes cell ballooning (arrowheads), immune cell infiltration (arrows), fibrosis (H&E), and fat accumulation detected with Oil red-O (ORO). Calibration bar, 20 µM. WD increases body weight **(B)**, liver weight **(C)** and liver/body weight ratio **(D)**, ORO **(E)**, triglycerides **(F)** and ALT **(G)**. All measures except weight gain are significantly reduced by AN1284 (1 mg/kg/day) and all except ALT, significantly reduced by AN1284 5 mg/kg/day. ANN12284 1 mg/kg/day restores ALT to normal by AN1284. Significantly different from ND, ***p < 0.001; significantly different from WD + saline, #p < 0.05; ##p < 0.01; ###p < 0.001.

During 4 months of feeding, mice on WD gained significantly more weight than those on the normal diet (*p* < 0.0001; **Figure 1B**). There were no significant differences in the weight gain between the three groups of mice on the WD. At this time, livers of mice on the WD showed significant hepatic fat content as showed with ORO staining (**Figure S1B**). During the last 2 months after implantation of the mps, there was still a significant difference in weight gain between saline-treated mice on a WD and those on an ND. AN1284 only significantly reduced weight gain at a dose 5 mg/kg/day (**Figure 1B**). After 4 months, the livers of saline-treated mice fed a WD showed extensive cell ballooning, inflammation (H&E), and fat accumulation (ORO) (**Figure 1A**). AN1284 (1 and 5 mg/kg/day) significantly decreased liver weight (**Figures 1C, D**), lipid content (**Figure 1E**), triglycerides (**Figure 1F**), and serum ALT (**Figure 1G**), despite the fact that they remained on the WD throughout the entire period. AN1284 also reduced hepatic cell ballooning and inflammation (**Figure 1A**). Additionally, we checked whether AN1284 has any effect in mice fed a ND. Neither dose of AN1284 had any significant effect on body weight, liver weight, ALT, and oil red content.

### 3.3 AN1284 attenuates liver fibrosis

**Figure 2:**
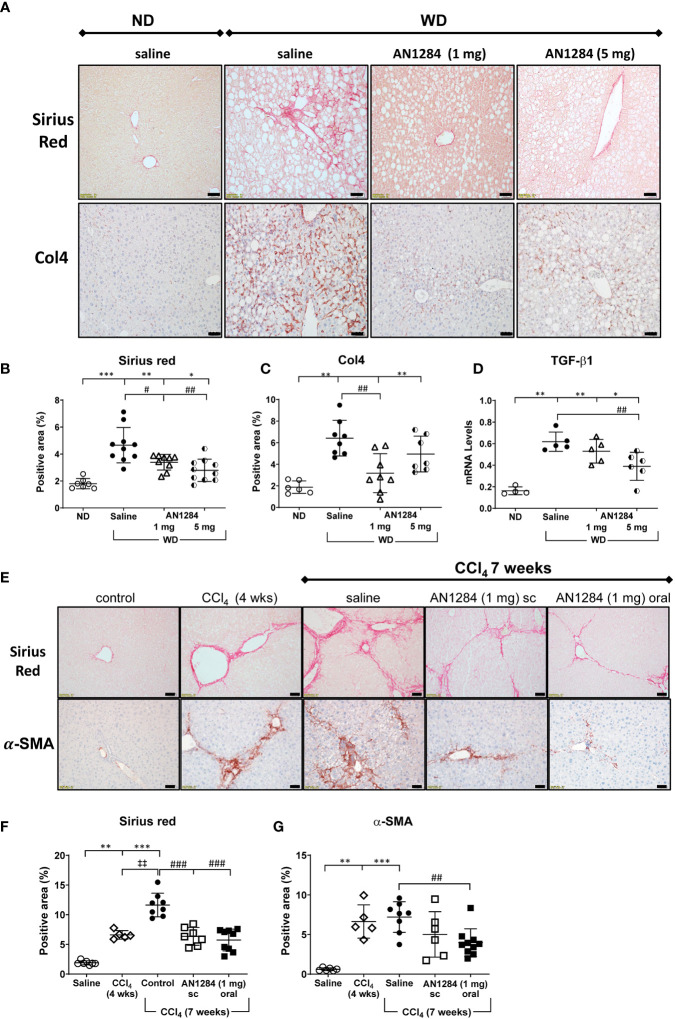
AN1284 reduces hepatic fibrosis in mice on a WD or after CCl4 injection. **(A)** WD increases the area of Sirius Red (SR) and Col4 and TGF-β1 compared to that in mice on ND. Calibration bar, 20 μM. **(B)** SR staining is significantly decreased by AN1284 (1 and 5 mg/kg/day). **(C)** Col4 staining is significantly decreased by AN1284 (1 mg/kg/day) but not by 5 mg/kg/day. **(D)** TGF-β1 mRNA is significantly reduced by AN1284 (5 mg/kg/day) but not by 1 mg/kg/day. **(E)** SR and α-SMA staining in mice is increased 4 and 7 weeks after bi-weekly injections of CCl4, a model of liver fibrosis. Calibration bar, 20 μM. **(F)** SR staining is significantly reduced by AN1284 1 mg/kg/day given by mps or in the drinking fluid. **(G)** α-SMA staining is significantly reduced by AN1284 given in the drinking fluid. Significantly different from control, *p < 0.05; **p < 0.01, ***p < 0.001. Significantly different from CCl4 4 weeks, ‡p < 0.01; significantly different from saline, #p < 0.05, ##p < 0.01, ###p < 0.001.

Moderate pericellular fibrosis in the livers of mice on the WD was demonstrated by an increase in staining with SR and Col4 and by TGF-β1 mRNA levels (**Figures 2A-D**). AN1284 (1 and 5 mg/kg/day) decreased SR. TGF-β1 mRNA was significantly reduced by 1 and 5 mg/kg/day but Col4 was significantly reduced only by a dose of 1 mg/kg/day. Since the degree of fibrosis was only moderate in the mice on WD, we performed additional experiments to assess the effect of AN1284 in mice on ND in which liver fibrosis was induced by injections of CCl_4_ during a 7-week period. Fibrosis, assessed by SR and α-SMA staining, was already present at 4 weeks (**Figure 2E**). SR intensity increased significantly by 7 weeks. AN1284 (1 mg/kg/day) given by mps or orally, started after 4 weeks of CCl_4_ injections when fibrosis was clearly present, decreased the levels of SR, but α-SMA was only reduced significantly after oral administration. The results indicate that AN1284 is able to halt the progression of liver fibrosis (**Figures 2E-G**).

### 3.4 AN1284 reverses hepatic gene expression related to liver diseases

**Figure 3:**
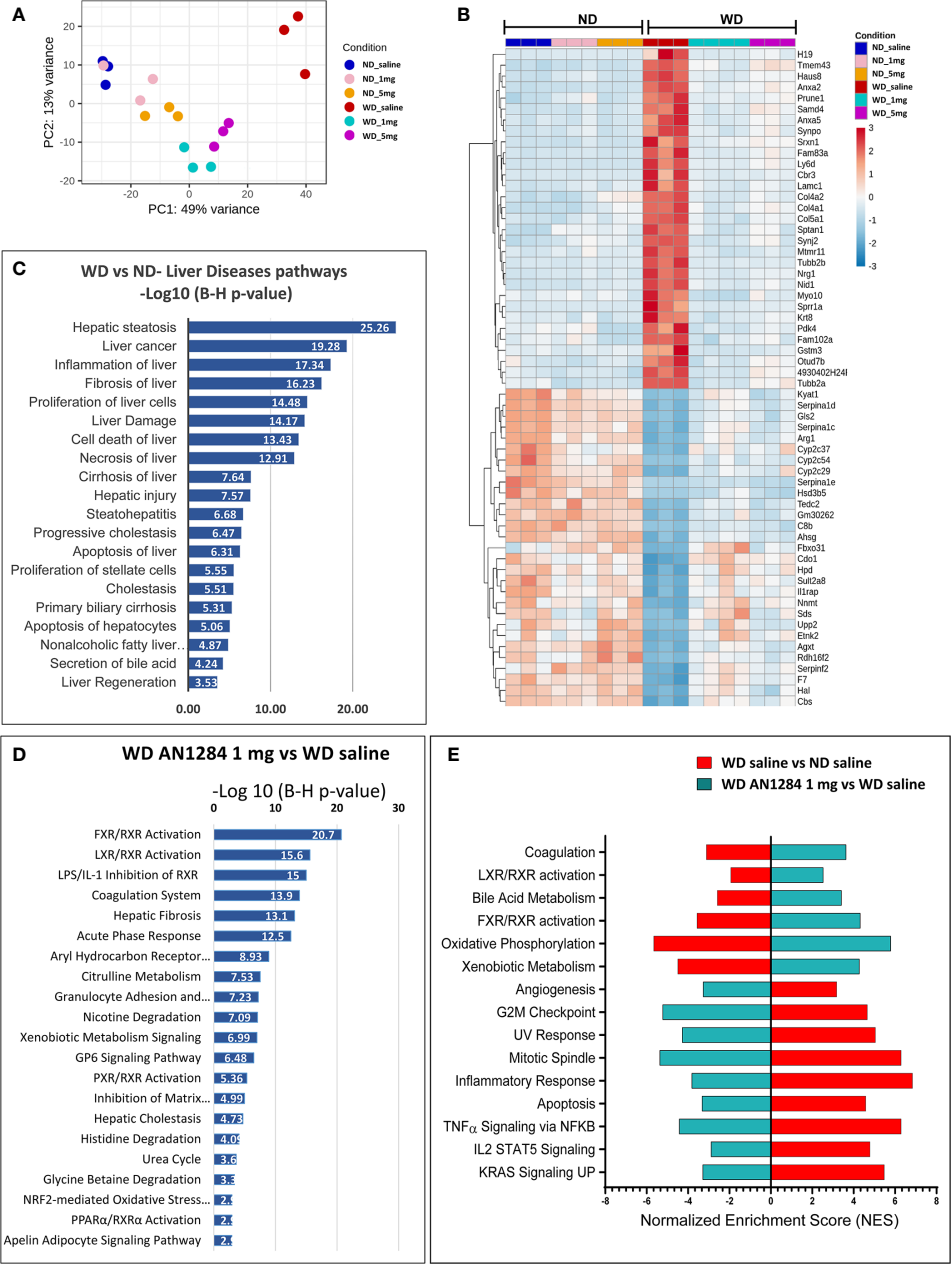
Effect of WD and AN1284 treatment on hepatic gene expression. **(A)** PCA plot showing RNA-Seq samples analyzed by diet and treatment groups. The WD substantially alters gene expression, while AN1284 treatment returns it to that of mice on a ND. **(B)** Heat map of genes most changed by the WD and the effect of AN1284 (1 and 5 mg/kg/day) on them. Red and blue colors indicate high and low gene expression, respectively. **(C)** IPA showing the top 20 pathways involved in liver disease and function that were significantly elevated by the WD compared to ND. Values are expressed as –log (B–H *p*-value). **(D)** IPA showing the top 20 canonical pathways that were significantly altered in AN1284-treated mice on a WD compared to those treated with saline. Values are expressed as –log (B–H *p*-value). **(E)** GSEA showing the most significant, enriched pathways that were up- or downregulated by the WD and the change reversed by AN1284 treatment.

We used RNA-Seq analysis to elucidate the influence of AN1284 on WD-induced hepatic gene expression profile. This enabled us to identify the canonical pathways altered by both the WD and drug treatment and to assess the differences in global gene expression between groups. Principal component analysis (PCA) showed that the six experimental groups could clearly be separated by the first two principal components (PC1 and PC2; **Figure 3A**). Compared to a ND, the WD significantly changed the expression of 4,600 genes [with a Base Mean (BM) >150]. Those most changed by the WD and reversed by AN1284 are shown in **Figure 3B**. IPA and GSEA also revealed the top 20 pathways significantly altered by the diet that are associated with liver diseases (**Figure 3C**). The WD strongly activated pathways of hepatic steatosis and fibrosis and those encoding inflammation, oxidative stress, liver damage, and liver necrosis. All were significantly altered by AN1284 treatment, together with liver metabolism and elevation of the (FXR)/retinoid X receptor (RXR) and liver X (LXR) receptors (**Figures 3D, E**). The xenobiotic metabolism and AhR pathways were also significantly altered by AN1284. IPA prediction of the up- or downstream regulators by AN1284 (**Supplementary Table 1**) indicated a role of several nuclear receptors (AhR, RXR, LXR, CAR, and FXR) and the inhibition of several cytokines and growth factors (i.e., TGF-β, TNF-α, IL-1β, and FGF). IPA pathways analysis suggested a decrease for AhR and an elevation of RXR and LXR (**Figures S2–S4**).

### 3.5 Effect of AN1284 on LXR/RXR and FXR/RXR pathways

**Figure 4:**
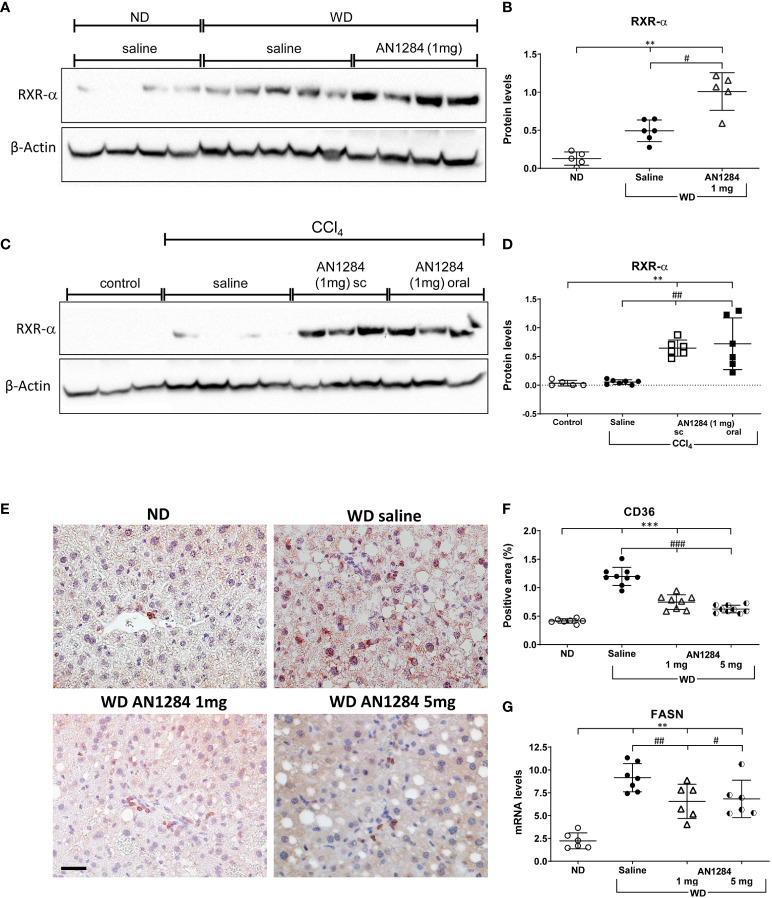
AN1284 increases RXR-α protein levels and decreases CD36 and FASN in mice on a WD. **(A, B)** WB of RXRα in mice on a WD. WD increases RXRα protein levels, which are further increased by AN1284 (1 mg/kg/day). The 5-mg dose was not tested. **(C, D)** WB of RXRα in mice after injection of CCl_4_. CCl_4_ alone has no effect on the levels of RXR-α, which were markedly increased by AN1284 (1 mg/kg/day) given by mps or in the drinking water. **(E, F)** Immunohistochemical staining of CD36-positive cells. The cells, indicated by red staining, are a significantly greater proportion of the area in mice on WD than on ND and are markedly reduced by AN1284 (1 and 5 mg/kg/day). **(G)** mRNA levels of FASN in mice on WD. FASN mRNA levels are increased by the WD and reduced by AN1284 (5 mg/kg/day). 1 mg/kg/day (*p* = 0.05). Significantly different from ND, **p < 0.01, ***p < 0.001; significantly different from WD + saline, #*p* < 0.05, ##*p* < 0.01, ###*p* < 0.001.

In recent years, the role of nuclear receptors in liver steatosis and NASH has been investigated. While some of them were initially characterized as xenobiotic receptors, subsequent observations have pointed to their equally important metabolic functions (27, 28). FXR and LXR control metabolic processes abundantly expressed in the liver. IPA and GSEAs indicated that AN1284 treatment activated the FXR/RXR pathway with a *p*-value of 20.7 and the LXR/RXR pathway with a *p*-value of 15.6 (**Figure 3D**, **S2**, **S3**). This was verified by WB analysis, which showed that levels of hepatic RXRα protein increased by the diet were further elevated by AN1284 (**Figures 4A, B**). Although RXRα protein levels did not change in the liver of mice, 7 weeks after CCl_4_ injections, they were greatly increased by both routes of AN1284 administration (**Figures 4C, D**). The WD also increased the percent area of fatty acid translocase (CD36)-positive cells (**Figures 4E, F**) and hepatic mRNA levels of FASN (**Figure 4G**) as suggested from RNA-Seq results (**Figure S2**). AN1284 (1 and 5 mg/kg/day) significantly decreased gene expression of CD36, ACC, and FASN.

### 3.6 AN1284 switches hepatic immune response from pro- to anti-inflammatory

**Figure 5:**
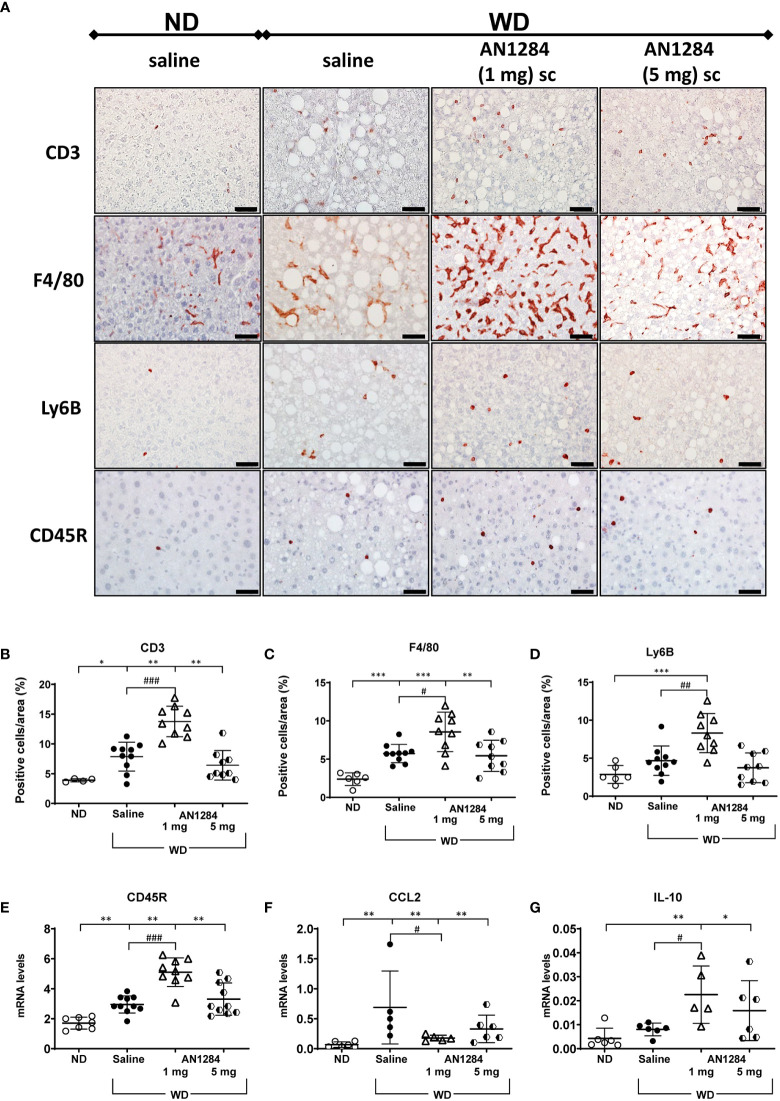
Effect of AN1284 on hepatic immune cells, anti-inflammatory cytokines, and CCL2 in mice on a WD. **(A–E)**. Immunohistochemical staining of immune cells in mice livers on a WD. CD3 for T cells, F4/80 for macrophages, CD45R for B cells, and Ly6B for neutrophils and NK cells. Calibration bar, 20 µM. **(F)**. mRNA levels of CCL2 in the liver of mice on a WD. WD increases T cells, macrophages and CCL2 expression. AN1284 (1 mg/kg/day) significantly further increases T cells **(B)**, macrophages **(C)**, neutrophils **(D)**, B cells **(E)** and IL-10 hepatic levels **(G)** but decreases CCL2 mRNA **(F)** Significantly different from ND, *p < 0.05; **p < 0.01; ***p < 0.001; significantly different from WD, #p < 0.05; ##p < 0.01; ###p < 0.001.

Hepatic inflammation plays an important role in the progression of NASH. Since the AhR is involved in many inflammatory responses, including suppression of cytokine release in LPS activated macrophages (28), we examined whether AN1284 also influences hepatic inflammation. In the livers of saline-treated mice, the WD significantly increased the number of hepatic T cells (CD3), macrophages (F4/80), and B cells (CD45R), but not neutrophils (Ly6B; **Figures 5A-E**). It also increased hepatic gene expression of CCL2 (**Figure 5F**), a marker of immune activation. AN1284 (1 mg/kg/day), but not 5 mg, increased the number of neutrophils and further increased that of macrophages, B cells, and T cells (**Figures 5A-E**). AN1284 depressed CCL2 gene expression (**Figure 5F**) and increased that of IL-10 (**Figure 5G**).

### 3.7 AN1284 reduces steatosis in isolated human hepatoma cells through AhR nuclear translocation

**Figure 6:**
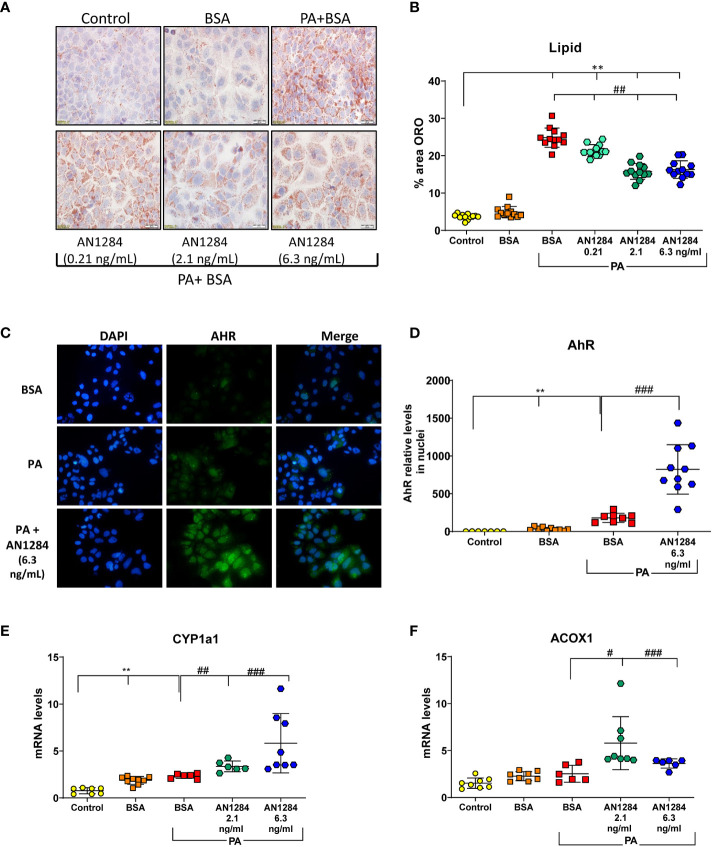
AN1284 decreases lipid generation from palmitic acid (PA), in human hepatoma cells in culture, through AhR activation. **(A)** Representative ORO staining in HUH7 human hepatoma cells incubated with PA/BSA and treated with increasing doses of AN1284 for 24 h. **(B)** Percent area of ORO in hepatoma cells after 2 h. **(C)** Nuclear translocation of AhR, 15 min after AN1284 addition. **(D)** AhR nuclear quantitation 15 min after AN1284 addition. **(E)** CYP1a1 mRNA levels after 24 h. **(F)** ACOX1 mRNA levels after 24 h. Significantly different from control **p < 0.01; significantly different from BSA + PA, #p < 0.05; ##p < 0.01; ###p < 0.001. These concentrations of AN1284 are within the range found in the liver after chronic treatment at 1 mg/kg/day by mps in this study.

**Figure 7:**
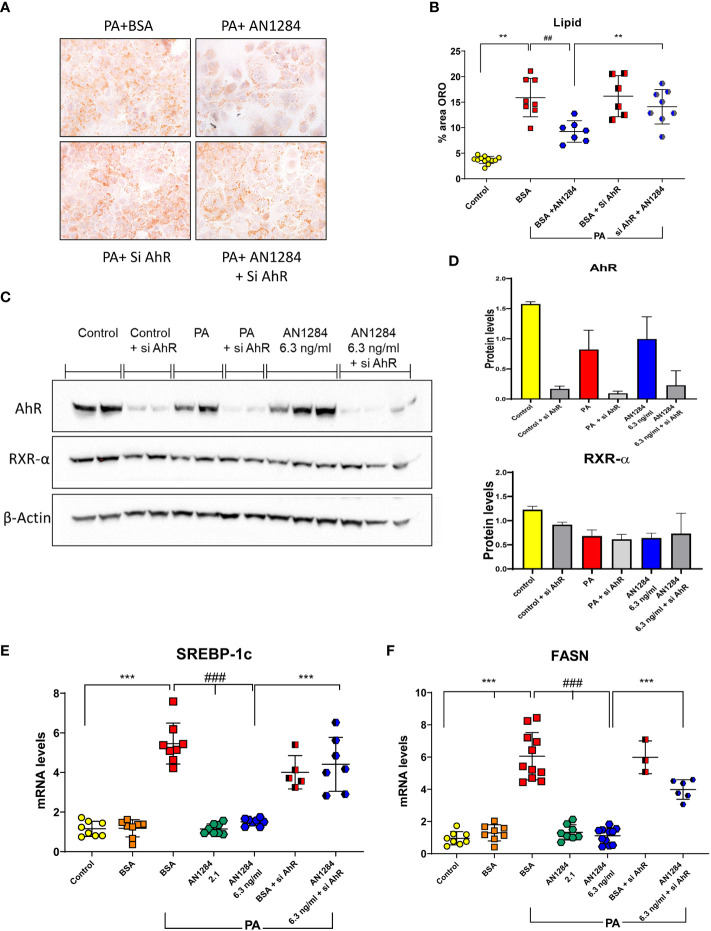
AhR silencing in human hepatoma cells by siRNA blocks the beneficial effects of AN1284 on lipid generation. **(A)** Representative ORO staining in HUH7 human hepatoma cells incubated with PA/BSA and treated with AN1284 ± siRNA for 24 h. **(B)** Percent area of ORO after 24 h. **(C)** Western blot of AhR and RXR-α proteins in HUH7 hepatoma cells incubated with PA and treated with AN1284 (6 ng/ml) ± siRNA for 24 h. **(D)** AhR silencing with siRNA reduced AhR protein levels in hepatoma cells but had no effect on RXR-α levels. **(E)** SREBP-1c mRNA levels after 24 h. **(F)** FASN mRNA levels after 24 h. Significantly different from control **p < 0.01; ***p < 0.001. Significantly different from BSA + PA, ##*p* < 0.01; ###*p* < 0.001.

Previous studies indicated that AhR acts as a “double-edged sword” in the progression of NAFLD, depending on the specific ligand (29). In order to determine whether AN1284 can have a direct effect on liver cells, we used a HUH7- human hepatoma cell line. The addition of PA/BSA complex to HUH7 cells for 48 h increased fat content (*p* < 0.0001). This was significantly reduced by AN1284 (0.21, 2.1, and 6.3 ng/ml) (**Figures 6A, B**). The concentrations were in the range of those found in the liver of mice treated with 1 and 5 mg/kg/day. BSA alone had no effect on the measurements. Since the RNA-Seq results suggested AhR as an upstream regulator, we analyzed its nuclear translocation in the HUH7 cells incubated with PA, 15 min after the addition of AN1284, and found this to be increased by AN1284 (6.3 ng/ml) (**Figures 6C, D**), together with upregulation in the expression of AhR target gene CYP1a1 and also ACOX1 (**Figures 6E, F**) 24 h later. AN1284 also decreased SREBP-1c mRNA, the principal transcriptional regulator of FASN that was elevated by PA (*p* < 0.001, **Figure 7E**). Similarly, FASN mRNA was decreased by AN1284 (2.1 and 6.3 ng/ml), opposing the increase caused by PA addition (*p* < 0.001; **Figure 7F**). In order to confirm that AN1284 suppresses fat accumulation in HUH7 cells through AhR, we silenced the receptor by using siRNA. AN1284 no longer reduced lipid in cells pre-treated with siRNA (**Figures 7A, B**). AhR protein levels were substantially reduced in HUH7 cells treated with AhR siRNA (**Figures 7C, D**). When these cells were incubated with PA and pre-treated with AN1284 siRNA, the levels of SREBP-1 and FASN genes remained elevated (**Figures 7E, F**). We also checked if AN1284 directly elevated RXR-α in the hepatoma cells. No change was observed in its protein levels in the WB analysis (**Figures 7C, D**).

## 4 Discussion

In our earlier study performed in diabetic mice (22), AN1284 was administered before the mice had any kidney or liver damage, in contrast to the current study in which drug treatment was only started after there were clear signs of hepatic steatosis and/or fibrosis. We now show that the WD given to mice for 4 months replicated much of the pathology in the liver of human subjects with NASH. It was supported by RNA-seq and IPA and GSEAs showing that the diet upregulated several of the major pathways affected in humans. These included hepatic steatosis, inflammation, fibrosis, hepatic cell proliferation, and oxidative stress. The findings were confirmed by direct measures of genes and proteins, which included significant increases in TGF-β1, Col4, and CD36, all of which are higher in humans with NASH (30–32). CD36 facilitates the intracellular uptake of FFAs and their esterification into triglycerides, while FASN catalyzes the last step in fatty acid biosynthesis and is believed to be a major determinant of lipogenesis (32, 33).

AN1284 (1 or 5 mg/kg/day), administered for 2 months by continuous release mps after commencement of the WD, reduced the deterioration of many of its deleterious effects, while the mice remained on the diet. This included the alterations in liver pathology, steatosis, and fibrosis and the percent of inducible nitric oxide synthase (iNOS)-positive cells (**Figure S5**), indicating that it was able to lower oxidative stress. AN1284 (1 mg/kg/day) also reduced the gene expression of pro-inflammatory factors TNF-α and CCL2 and increased that of IL-10. CCL2 promotes fibrosis by recruiting pro-inflammatory monocytes (34). In the later, resolution stage of NASH, macrophages change their phenotype, expressing cytokines like IL-10 that suppress the proliferation and effector functions of CD4^+^ and CD8^+^ T cells and repair wound healing (35). AN1284 decreased SREBP-1c mRNA, while increasing that of ACOX-1, an enzyme found in peroxisomes and mitochondria, which oxidizes straight chain fatty acids like PA. Other studies have shown that inhibition of ACOX-1 or an abnormal ACOX-1 gene (36) can increase steatosis.

Numerous nuclear receptors including FXR, LXR, RXR, and AhR have been suggested as regulators of NAFLD and NASH progression (27, 28). RNA-Seq analysis points to the involvement of these nuclear receptors in the mechanism of action of AN1284. RXRα is a nuclear receptor that forms a heterodimer with other such receptors like FXR, LXR, and PPAR to promote cholesterol efflux. It helps to regulate glucose metabolism, apoptotic cell clearance, immune cell proliferation, and inflammatory gene repression (37). FXR is reduced in patients with NASH (38), and its levels of expression are inversely correlated with disease severity (39). When given either by mps or in the drinking water, AN1284 activated the FXR–RXR pathway and increased the levels of RXRα protein in mice on the WD and also in those with fibrosis, induced by CCl_4_ injections.

Using RNA-Seq to elucidate the mechanism of action of AN1284, we found that it reduced AhR mRNA levels and activated genes downstream of AhR. AhR signaling appears to be involved in immune-mediated diseases in humans (40). Depending on the particular cell type and the activating ligands, AhR was reported to have an anti-inflammatory and tissue-protective function in immune-mediated liver disease (41). Yet, the role of AhR in NAFLD remains controversial and appears to depend on the model used. In mice with constitutively activated human AhR given a WD, the level of steatosis was higher than in controls (42). However, stimulation of AhR with indole propionic acid, which shares some of the anti-inflammatory activity of indolines, but at higher concentrations (43), alleviated steatosis in mice on a WD (44). Moreover, activation of AhR in hepatic stellate cells prevented fibrosis induced by CCl_4_ injections by blocking downstream genes required for fibrogenesis (45). Additionally, AhR was shown to play a role in the regulation of body mass in mice fed a WD (46). In a previous study, on db/db mice, AN1284 arrested body weight gain at a dose of 5 mg/kg/day only after 2 months of treatment and significantly increased total body fat oxidation (22). In the current study, both doses of AN1284 attenuated liver weight, but body weight gain was again only significantly decreased by a dose of 5 mg/kg/day.

In a human hepatoma cell line incubated with PA, we found that AhR was translocated to the nucleus 15 min following the administration of AN1284. The expression of CYP1a1 gene downstream of AhR, was upregulated 24 h later, but AN1284 had no direct effect on protein levels of AhR and RXR-α (**Figures 7C, D**). On the other hand, genes related to the LXR pathway, FASN and SREBP-1c, were significantly reduced by AN1284 (2.1 and 6.3 ng/ml). Silencing AhR in the hepatoma cells confirmed that part of the direct actions of AN1284 is mediated through AhR activation.

While one or the other of the two doses of AN1284 given in this study appeared to be more effective in altering some measures of liver pathology, there were no statistically significant differences between any of their effects. Neither did they produce significant differences in hepatic concentrations, but those of the indole metabolite were higher after 5 mg/kg/day. Although not measured in the current study, the hepatic concentrations of AN1284 after administration of 1 mg/kg/day in the drinking water that significantly reduced fibrosis in the CCl_4_ model were similar to those achieved by administration of 2.5 mg/kg/day by mps (25).

In conclusion, AN1284 given to mice for 2 months at doses of 1 and 5 mg/kg/day can mitigate the deterioration of hepatic damage, steatosis, and fibrosis caused by a modified WD, in part through the AhR nuclear receptor that controls several, independent processes that were shown to promote NASH in human subjects. The beneficial effect of AN1284 on liver pathology in NASH may be due to a combination of a reduction in liver weight, inflammation, oxidative stress, and fibrosis.

## Data availability statement

The datasets presented in this study can be found in online repositories. The names of the repository/repositories and accession number(s) can be found below: GSE186116 (GEO).

## Ethics statement

The animal study was reviewed and approved by Experiments were performed according to the guidelines of the Animal Care and Use Committee of the Hebrew University (NIH approval number OPRR-A01-5011).

## Author contributions

RA and MW contributed to conception and design of the study. ASY, NA, and RA performed the experiments. ASY, RA, and MW organized the database. SE, HB, and YN performed the bioinformatics analysis. MW and RA wrote the manuscript. All authors contributed to manuscript revision, and read and approved the submitted version.

## References

[1] Enomoto H Bando Y Nakamura H Nishiguchi S Koga M. Liver fibrosis markers of nonalcoholic steatohepatitis. World J Gastroenterol (2015) 21:7427–35. doi: 10.3748/wjg.v21.i24.7427 PMC4481437 26139988

[2] Spahis S Delvin E Borys JM Levy E. Oxidative stress as a critical factor in nonalcoholic fatty liver disease pathogenesis. Antioxid Redox Signal (2017) 26:519–41. doi: 10.1089/ars.2016.6776 27452109

[3] Marra F Svegliati-Baroni G. Lipotoxicity and the gut-liver axis in NASH pathogenesis. J Hepatol (2018) 68:280–95. doi: 10.1016/j.jhep.2017.11.014 29154964

[4] Xiang M Wang PX Wang AB Zhang XJ Zhang Y Zhang P. Targeting hepatic TRAF1-ASK1 signaling to improve inflammation, insulin resistance, and hepatic steatosis. J Hepatol (2016) 64:1365–77. doi: 10.1016/j.jhep.2016.02.002 26860405

[5] Liu RM Desai LP. Reciprocal regulation of TGF-beta and reactive oxygen species: A perverse cycle for fibrosis. Redox Biol (2015) 6:565–77. doi: 10.1016/j.redox.2015.09.009 PMC4625010 26496488

[6] Caesar R Tremaroli V Kovatcheva-Datchary P Cani PD Backhed F. Crosstalk between Gut Microbiota and Dietary Lipids Aggravates WAT Inflammation through TLR Signaling. Cell Metab (2015) 22:658–68. doi: 10.1016/j.cmet.2015.07.026 PMC4598654 26321659

[7] Dulai PS Singh S Patel J Soni M Prokop LJ Younossi Z. Increased risk of mortality by fibrosis stage in nonalcoholic fatty liver disease: Systematic review and meta-analysis. Hepatology (2017) 65:1557–65. doi: 10.1002/hep.29085 PMC5397356 28130788

[8] Carpino G Del Ben M Pastori D Carnevale R Baratta F Overi D. Increased liver localization of lipopolysaccharides in human and experimental NAFLD. Hepatology (2020) 72:470–85. doi: 10.1002/hep.31056 31808577

[9] Bao L Yin J Gao W Wang Q Yao W Gao X. A long-acting FGF21 alleviates hepatic steatosis and inflammation in a mouse model of non-alcoholic steatohepatitis partly through an FGF21-adiponectin-IL17A pathway. Br J Pharmacol (2018) 175:3379–93. doi: 10.1111/bph.14383 PMC6057909 29859019

[10] Kim MH Kang KS Lee YS. The inhibitory effect of genistein on hepatic steatosis is linked to visceral adipocyte metabolism in mice with diet-induced non-alcoholic fatty liver disease. Br J Nutr (2010) 104:1333–42. doi: 10.1017/S0007114510002266 20687969

[11] Park EJ Kim YM Kim HJ Jang SY Oh MH Lee DH. (S)YS-51, a novel isoquinoline alkaloid, attenuates obesity-associated non-alcoholic fatty liver disease in mice by suppressing lipogenesis, inflammation and coagulation. Eur J Pharmacol (2016) 788:200–9. doi: 10.1016/j.ejphar.2016.06.040 27343380

[12] Romero-Zerbo SY Garcia-Fernandez M Espinosa-Jimenez V Pozo-Morales M Escamilla-Sanchez A Sanchez-Salido L. The atypical cannabinoid abn-CBD reduces inflammation and protects liver, pancreas, and adipose tissue in a mouse model of prediabetes and non-alcoholic fatty liver disease. Front Endocrinol (Lausanne) (2020) 11:103. doi: 10.3389/fendo.2020.00103 32210914 PMC7067697

[13] Yao Q Li S Li X Wang F Tu C. Myricetin modulates macrophage polarization and mitigates liver inflammation and fibrosis in a murine model of nonalcoholic steatohepatitis. Front Med (Lausanne) (2020) 7:71. doi: 10.3389/fmed.2020.00071 32195263 PMC7065264

[14] Patil NY Rus I Downing E Mandala A Friedman JE Joshi AD. Cinnabarinic acid provides hepatoprotection against nonalcoholic fatty liver disease. J Pharmacol Exp Ther (2022) 383:32–43. doi: 10.1124/jpet.122.001301 35933113 PMC9513857

[15] Gapp B Jourdain M Bringer P Kueng B Weber D Osmont A. Farnesoid X receptor agonism, acetyl-coenzyme A carboxylase inhibition, and back translation of clinically observed endpoints of *de novo* lipogenesis in a murine NASH model. Hepatol Commun (2020) 4:109–25. doi: 10.1002/hep4.1443 PMC6939503 31909359

[16] Alkhouri N Lawitz E Noureddin M DeFronzo R Shulman GI. GS-0976 (Firsocostat): an investigational liver-directed acetyl-CoA carboxylase (ACC) inhibitor for the treatment of non-alcoholic steatohepatitis (NASH). Expert Opin Investig Drugs (2020) 29:135–41. doi: 10.1080/13543784.2020.1668374 PMC7063378 31519114

[17] Kaul U Parmar D Manjunath K Shah M Parmar K Patil KP. New dual peroxisome proliferator activated receptor agonist-Saroglitazar in diabetic dyslipidemia and non-alcoholic fatty liver disease: integrated analysis of the real world evidence. Cardiovasc Diabetol (2019) 18:80. doi: 10.1186/s12933-019-0884-3 31208414 PMC6580520

[18] Boeckmans J Natale A Rombaut M Buyl K Rogiers V De Kock J. Anti-NASH drug development hitches a lift on PPAR agonism. Cells (2020) 9(1):37. doi: 10.3390/cells9010037 PMC7016963 31877771

[19] Dufour JF Anstee QM Bugianesi E Harrison S Loomba R Paradis V. Current therapies and new developments in NASH. Gut (2022) 71:2123–34. doi: 10.1136/gutjnl-2021-326874 PMC9484366 35710299

[20] Zeeli S Weill T Finkin-Groner E Bejar C Melamed M Furman S. Synthesis and biological evaluation of derivatives of indoline as highly potent antioxidant and anti-inflammatory agents. J Med Chem (2018) 61:4004–19. doi: 10.1021/acs.jmedchem.8b00001 29681148

[21] Finkin-Groner E Finkin S Zeeli S Weinstock M. Indoline derivatives mitigate liver damage in a mouse model of acute liver injury. Pharmacol Rep (2017) 69:894–902. doi: 10.1016/j.pharep.2017.03.025 28628850

[22] Permyakova A Gammal A Hinden L Weitman M Weinstock M Tam J. A novel indoline derivative ameliorates diabesity-induced chronic kidney disease by reducing metabolic abnorMalities. Front Endocrinol (Lausanne) (2020) 11:91. doi: 10.3389/fendo.2020.00091 32218769 PMC7078689

[23] Della Torre S. Non-alcoholic fatty liver disease as a canonical example of metabolic inflammatory-based liver disease showing a sex-specific prevalence: relevance of estrogen signaling. Front Endocrinol (Lausanne) (2020) 11:572490. doi: 10.3389/fendo.2020.572490 33071979 PMC7531579

[24] Farrell G Schattenberg JM Leclercq I Yeh MM Goldin R Teoh N. Mouse models of nonalcoholic steatohepatitis: toward optimization of their relevance to human nonalcoholic steatohepatitis. Hepatology (2019) 69:2241–57. doi: 10.1002/hep.30333 30372785

[25] Weitman M Bejar C Melamed M Weill T Yanovsky I Zeeli S. Comparison of the tissue distribution and metabolism of AN1284, a potent anti-inflammatory agent, after subcutaneous and oral administration in mice. Naunyn Schmiedebergs Arch Pharmacol (2021) 394(10):2077–89. doi: 10.1007/s00210-021-02125-y 34309687

[26] Folch J Lees M Sloane SGH. A simple method for the isolation and purification of total lipides from animal tissues. J Biol Chem (1957) 226:497–509. doi: 10.1016/S0021-9258(18)64849-5 13428781

[27] Wang J Lu P Xie W. Atypical functions of xenobiotic receptors in lipid and glucose metabolism. Med Rev (2022) 2:611–24. doi: 10.1515/mr-2022-0032 PMC9912049 36785576

[28] Puengel T Liu H Guillot A Heymann F Tacke F Peiseler M. Nuclear receptors linking metabolism, inflammation, and fibrosis in nonalcoholic fatty liver disease. Int J Mol Sci (2022) 23:2668. doi: 10.3390/ijms23052668 35269812 PMC8910763

[29] Nguyen NT Hanieh H Nakahama T Kishimoto T. The roles of aryl hydrocarbon receptor in immune responses. Int Immunol (2013) 25:335–43. doi: 10.1093/intimm/dxt011 23580432

[30] Patil NY Friedman JE Joshi AD. Role of hepatic aryl hydrocarbon receptor in non-alcoholic fatty liver disease. Receptors (2023) 2:1–15. doi: 10.3390/receptors201000 37284280 PMC10240927

[31] Che L Paliogiannis P Cigliano A Pilo MG Chen X Calvisi DF. Pathogenetic, prognostic, and therapeutic role of fatty acid synthase in human hepatocellular carcinoma. Front Oncol (2019) . 9:1412. doi: 10.3389/fonc.2019.01412 31921669 PMC6927283

[32] Dorn C Riener MO Kirovski G Saugspier M Steib K Weiss TS. Expression of fatty acid synthase in nonalcoholic fatty liver disease. Int J Clin Exp Pathol (2010) 3:505–14. PMC2897101 20606731

[33] Rada P Gonzalez-Rodriguez A Garcia-Monzon C Valverde AM. Understanding lipotoxicity in NAFLD pathogenesis: is CD36 a key driver? Cell Death Dis (2020) 11:802. doi: 10.1038/s41419-020-03003-w 32978374 PMC7519685

[34] Ehling J Bartneck M Wei X Gremse F Fech V Mockel D. CCL2-dependent infiltrating macrophages promote angiogenesis in progressive liver fibrosis. Gut (2014) 63:1960–71. doi: 10.1136/gutjnl-2013-306294 PMC4216733 24561613

[35] Wen Y Lambrecht J Ju C Tacke F. Hepatic macrophages in liver homeostasis and diseases-diversity, plasticity and therapeutic opportunities. Cell Mol Immunol (2021) 18:45–56. doi: 10.1038/s41423-020-00558-8 33041338 PMC7852525

[36] Moreno-Fernandez ME Giles DA Stankiewicz TE Sheridan R Karns R Cappelletti M. Peroxisomal beta-oxidation regulates whole body metabolism, inflammatory vigor, and pathogenesis of nonalcoholic fatty liver disease. JCI Insight (2018) 3(6):e93626. doi: 10.1172/jci.insight.93626 PMC5926941 29563328

[37] Hiebl V Ladurner A Latkolik S Dirsch VM. Natural products as modulators of the nuclear receptors and metabolic sensors LXR, FXR and RXR. Biotechnol Adv (2018) 36:1657–98. doi: 10.1016/j.bioteChadv.2018.03.003 29548878

[38] Cariello M Piccinin E Moschetta A. Transcriptional regulation of metabolic pathways via lipid-sensing nuclear receptors PPARs, FXR, and LXR in NASH. Cell Mol Gastroenterol Hepatol (2021) 11:1519–39. doi: 10.1016/j.jcmgh.2021.01.012 PMC8042405 33545430

[39] Yang ZX Shen W Sun H. Effects of nuclear receptor FXR on the regulation of liver lipid metabolism in patients with non-alcoholic fatty liver disease. Hepatol Int (2010) 4:741–8. doi: 10.1007/s12072-010-9202-6 PMC2994619 21286345

[40] Gutierrez-Vazquez C Quintana FJ. Regulation of the immune response by the aryl hydrocarbon receptor. Immunity (2018) 48:19–33. doi: 10.1016/j.immuni.2017.12.012 29343438 PMC5777317

[41] Carambia A Schuran FA. The aryl hydrocarbon receptor in liver inflammation. Semin Immunopathol (2021) 43:563–75. doi: 10.1007/s00281-021-00867-8 PMC8443474 34075438

[42] Lu P Yan J Liu K Garbacz WG Wang P Xu M. Activation of aryl hydrocarbon receptor dissociates fatty liver from insulin resistance by inducing fibroblast growth factor 21. Hepatology (2015) 61:1908–19. doi: 10.1002/hep.27719 PMC4441569 25614121

[43] Furman S Nissim-Bardugo E Zeeli S Weitman M Nudelman A Finkin-Groner E. Synthesis and in *vitro* evaluation of anti-inflammatory activity of ester and amine derivatives of indoline in RAW 264.7 and peritoneal macrophages. Bioorg Med Chem Lett (2014) 24:2283–7. doi: 10.1016/j.bmcl.2014.03.081 24731278

[44] Xu X Sun S Liang L Lou C He Q Ran M. Role of the aryl hydrocarbon receptor and gut microbiota-derived metabolites indole-3-acetic acid in sulforaphane alleviates hepatic steatosis in mice. Front Nutr (2021) 8:756565. doi: 10.3389/fnut.2021.756565 34722615 PMC8548612

[45] Yan J Tung HC Li S Niu Y Garbacz WG Lu P. Aryl hydrocarbon receptor signaling prevents activation of hepatic stellate cells and liver fibrogenesis in mice. Gastroenterology (2019) 157:793–806 e714. doi: 10.1053/j.gastro.2019.05.066 31170413 PMC6707837

[46] Moyer BJ Rojas IY Kerley-Hamilton JS Hazlett HF Nemani KV Trask HW. Inhibition of the aryl hydrocarbon receptor prevents Western diet-induced obesity. Model for AHR activation by kynurenine via oxidized-LDL, TLR2/4, TGFβ, and IDO1. Toxicol Appl Pharmacol (2016) 300:13–24. doi: 10.1016/j.taap.2016.03.011 27020609 PMC4851598

