# Supplementary material for: AN1284 attenuates steatosis, lipogenesis, and fibrosis in mice with pre-existing non-alcoholic steatohepatitis and directly affects aryl hydrocarbon receptor in a hepatic cell line
Source: Front Endocrinol (Lausanne). 2023 Aug 16;14:1226808. doi: 10.3389/fendo.2023.1226808 (PMC10469006; doi:10.3389/fendo.2023.1226808)
Supplement: Supplementary file 1 [file Image_1.pdf]

Supplementary Figures

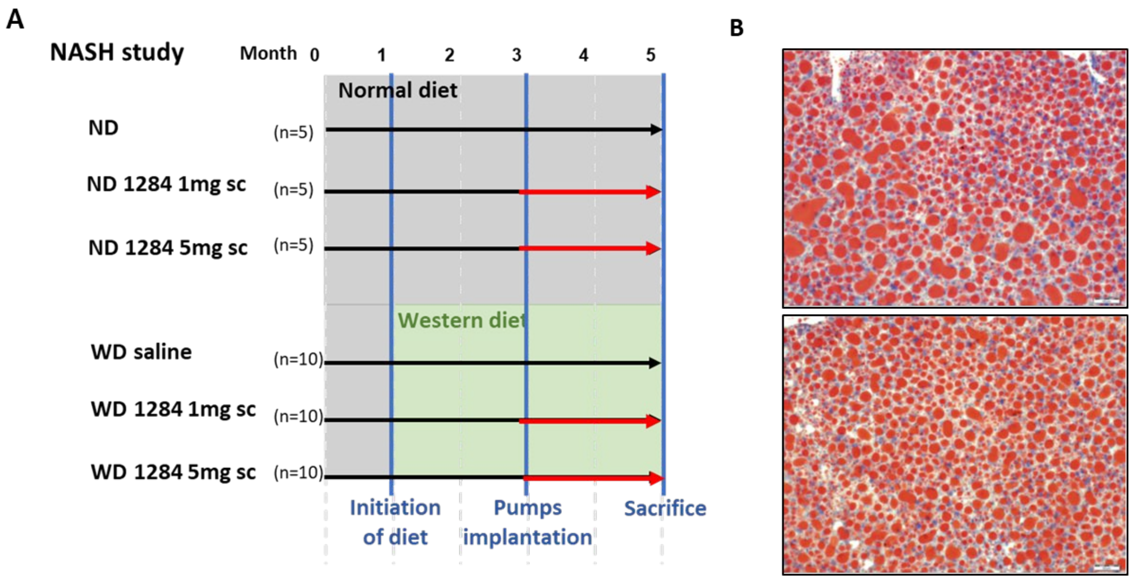

Fig. S1: Experimental protocol of WD feeding in mice and its effect on liver steatosis and body weight.
A) Experimental design and feeding protocol.
B) WD causes hepatic fat accumulation already after two months. Two representative samples stained with ORO.

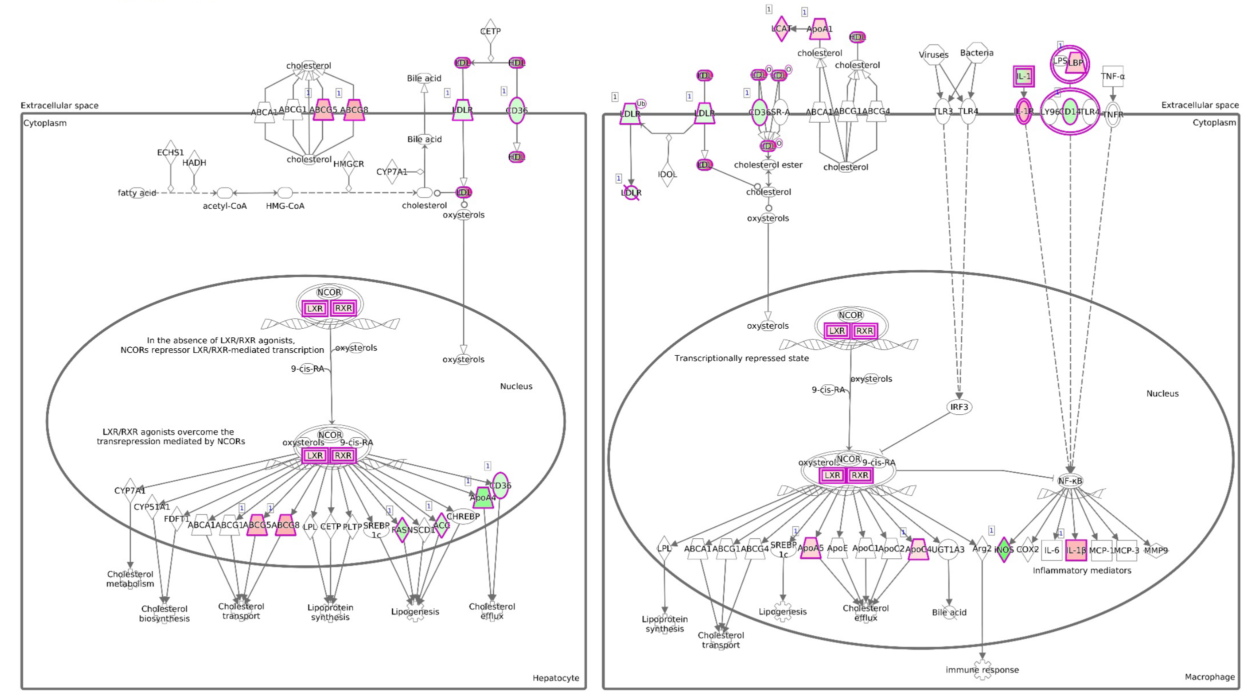

© 2000-2019 QIAGEN. All rights reserved.

<u>Fig. S2</u>: LXR/RXR pathway identified by Ingenuity pathway analysis (IPA)

The LXR/RXR pathway is identified by IPA as one of the significant pathways that is altered in mice on a WD given saline and those treated with AN1284. All the up-regulated genes are marked in red while those down-regulated are marked in green.

Nodes represent molecules in a pathway, while the biological relationship between nodes is represented by a line (edge). Edges are supported by at least one reference in the Ingenuity Knowledge Base. The intensity of color in a node indicates the degree of up- (red) or down- (green) regulation. Nodes are displayed using shapes that represent the functional class of a gene product (Circle = Other, Nested Circle = Group or Complex, Rhombus = Peptidase, Square = Cytokine, Triangle = Kinase, Vertical ellipse = Transmembrane receptor). Edges are marked with symbols to represent the relationship between nodes (Line only = Binding only, Flat line = inhibits, Solid arrow = Acts on, Solid arrow with flat line = inhibits and acts on, Open circle = leads to, Open arrow = translocates to).

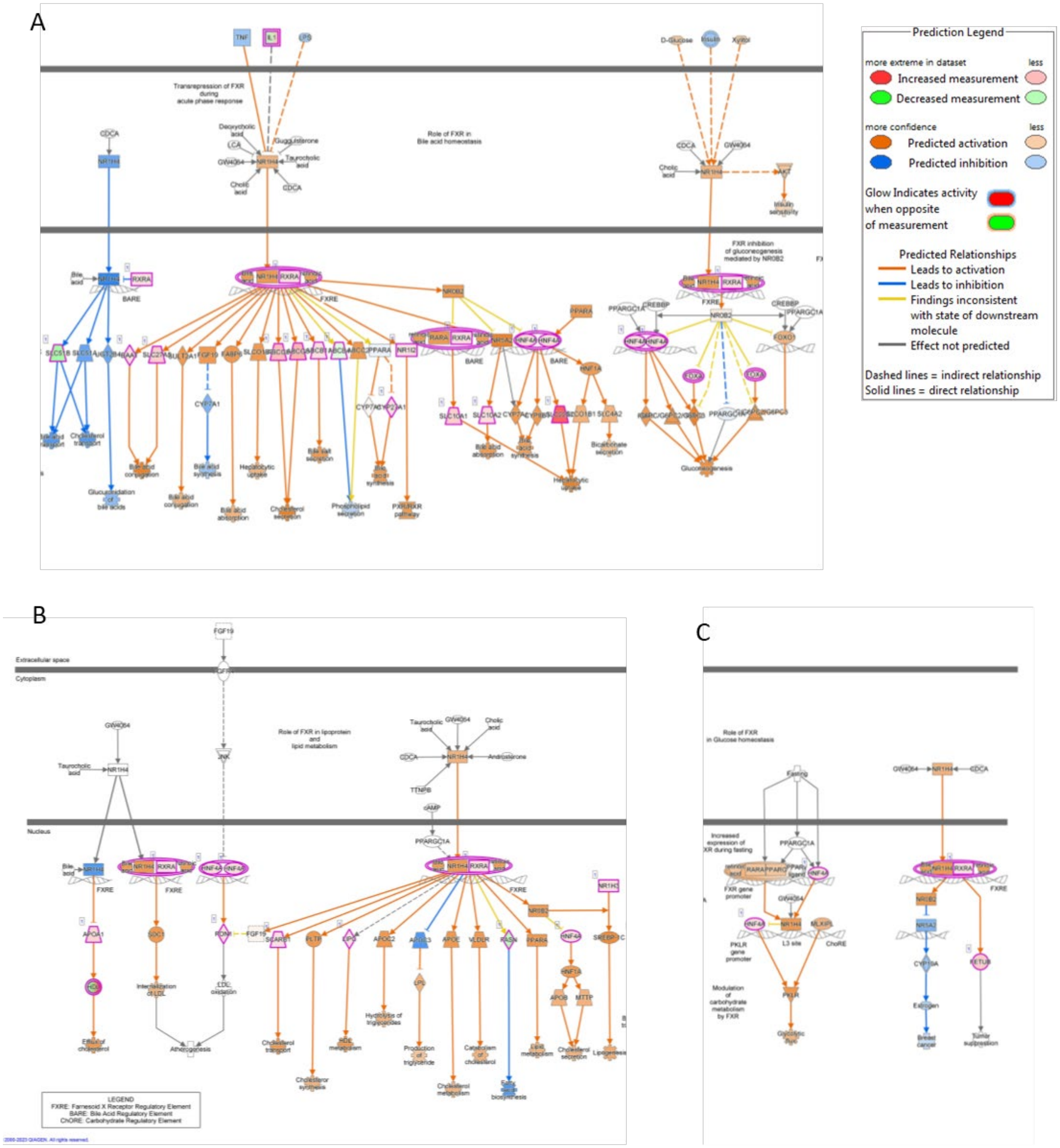

Fig. S3: FXR/RXR pathway identified by IPA

The FXR/RXR pathway is identified by IPA as one of the significant pathways that is altered in mice on a WD given saline and those treated with AN1284. A) Role of FXR in bile acid homeostasis; B) Role of FXR in lipid metabolism; C) Role of FXR in glucose homeostasis. All the significantly up-regulated genes are shaded in red while those down-regulated are shaded in green. Blue shading indicates predicted repression and orange shading indicates predicted elevation.

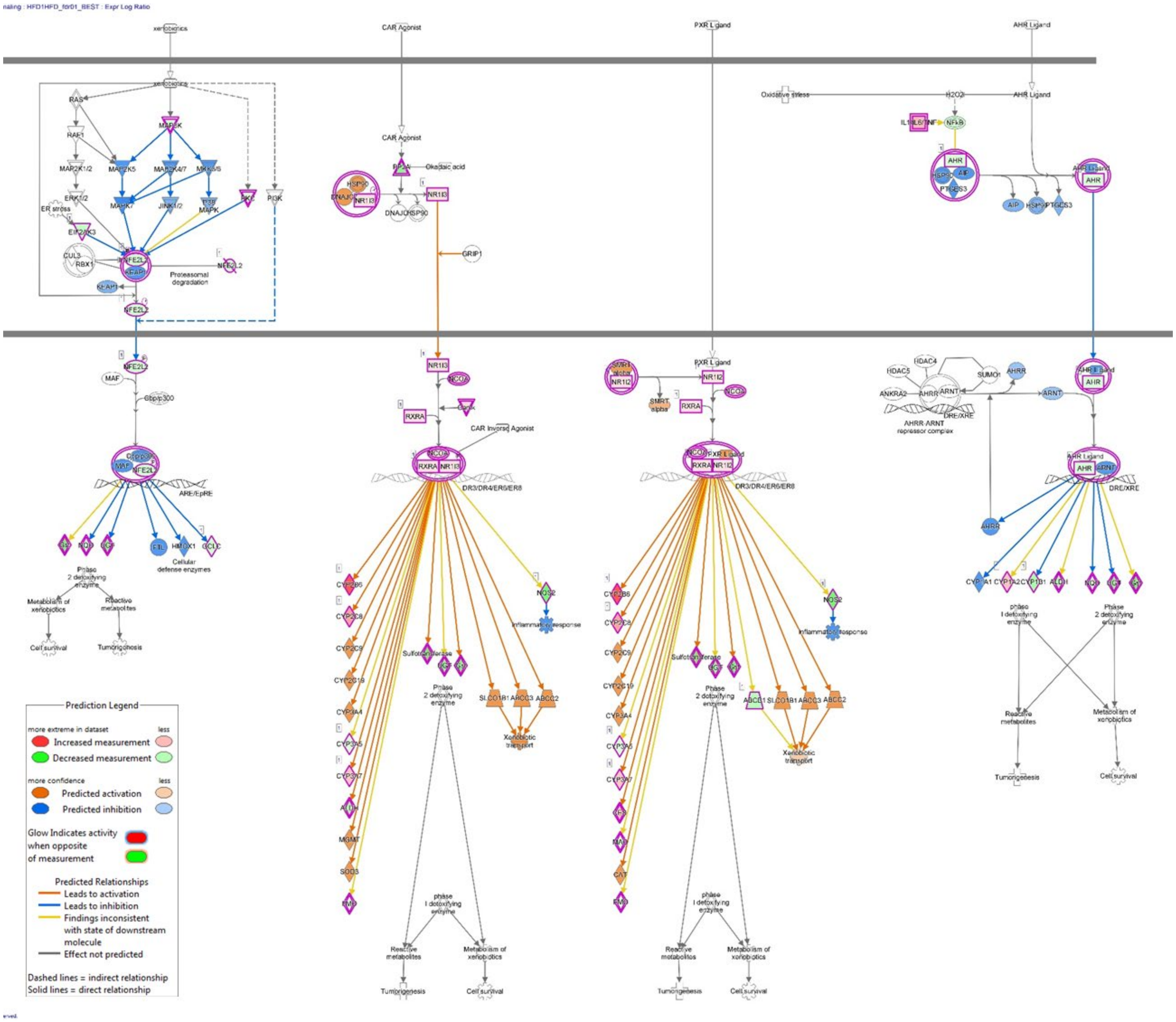

Fig. S4: Lipid xenobiotic pathway identified by IPA

The lipid xenobiotic pathway is identified by IPA as another one of the significant pathways that differ in mice on WD treated with AN1284 compared to WD and saline. All the significantly up-regulated genes are shaded in red while those down-regulated are shaded in green. Blue shading indicates predicted repression and orange shading indicates predicted elevation.

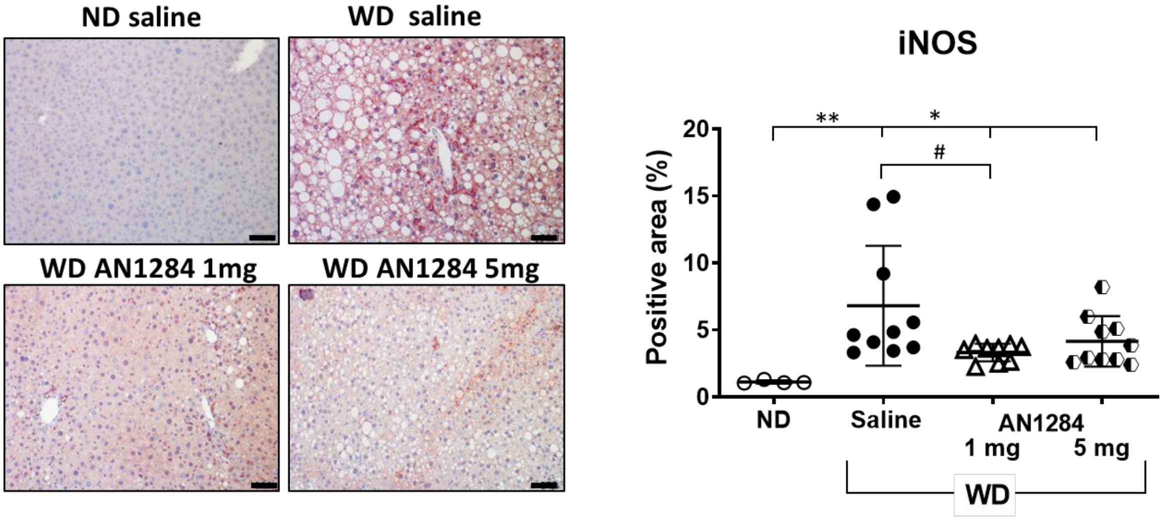

Fig. S5. Effect of AN1284 on oxidative stress in mice on a WD.

Immuno-histochemical staining of iNOS in liver of mice on ND and WD. WD significantly increases iNOS, which is reduced by AN1284. Significantly different from ND **, P<0.01; significantly different from WD + saline, #, P<0.05.
