# Supplementary material for: AN1284 attenuates steatosis, lipogenesis, and fibrosis in mice with pre-existing non-alcoholic steatohepatitis and directly affects aryl hydrocarbon receptor in a hepatic cell line
Source: Front Endocrinol (Lausanne). 2023 Aug 16;14:1226808. doi: 10.3389/fendo.2023.1226808 (PMC10469006; doi:10.3389/fendo.2023.1226808)
Supplement: Supplementary file 2 [file Table_1.pdf]

Upstream Regulator	Molecule Type	Predicted Activation State	Activation z-score	p-value	Log 10(p-value)
ACOX1	enzyme	Activated	7.319	1.71E-49	48.7670
AHR	ligand-dependent nuclear rece	Activated	2.693	5.18E-47	46.2857
HNF1A	transcription regulator	Activated	3.146	5.53E-28	27.2573
PPARGC1A	transcription regulator	Activated	3.205	2.15E-26	25.6676
NR3C1	ligand-dependent nuclear rece	Activated	3.31	1.9E-22	21.7212
estrogen receptor	group	Activated	2.992	1.22E-21	20.9136
STAT5B	transcription regulator	Activated	3.239	1.65E-21	20.7825
POR	enzyme	Activated	2.114	4.95E-20	19.3054
NR1H4 (FXR gene)	ligand-dependent nuclear rece	Activated	2.223	4.28E-18	17.3686
SMAD7	transcription regulator	Activated	2.968	6.64E-18	17.1778
SMARCB1	transcription regulator	Activated	2.966	8.9E-17	16.0506
ABCB4 (MDR3)	transporter	Activated	4.202	1.45E-15	14.8386
HNF4A	transcription regulator	Activated	5.705	1.29E-14	13.8894
CLOCK	transcription regulator	Activated	2.548	4.89E-12	11.3107
INSIG1	other	Activated	2.469	1.17E-10	9.9318
ALDH1A2	enzyme	Activated	2.191	2.87E-10	9.5421
TAF4	transcription regulator	Activated	2.533	3.58E-10	9.4461
FBN1	other	Activated	3.42	3.91E-10	9.4078
PKD1	ion channel	Activated	3.583	4.86E-10	9.3134
RUNX3	transcription regulator	Activated	3.51	1.35E-09	8.8697
AMPK	complex	Activated	2.563	1.22E-07	6.9136
GLIS2	transcription regulator	Activated	3.148	1.86E-07	6.7305
mir-29	microRNA	Activated	2.279	0.00000039	6.4089
miR-199a-3p	mature microRNA	Activated	3.59	5.49E-07	6.2604
GCG	other	Activated	2.253	0.00000567	5.2464
FOXA1	transcription regulator	Activated	2.167	0.00000753	5.1232
Irgm1	other	Activated	2.536	0.000024	4.6198
TRIM24	transcription regulator	Activated	2.668	0.000217	3.6635
FDFT1	enzyme	Activated	2.646	0.000246	3.6091
TGFB1	growth factor	Inhibited	-6.645	4.36E-57	56.3605
TNF	cytokine	Inhibited	-4.856	8.59E-48	47.0660
ERBB2	kinase	Inhibited	-2.718	1.17E-41	40.9318
PPARA	ligand-dependent nuclear rece	Inhibited	-2.755	1.62E-35	34.7905
IL1B	cytokine	Inhibited	-3.589	1.41E-31	30.8508
EGF	growth factor	Inhibited	-3.745	1.16E-25	24.9355
CCR2	G-protein coupled receptor	Inhibited	-4.064	3.82E-24	23.4179
Tgf beta	group	Inhibited	-4.03	9.89E-24	23.0048
OSM	cytokine	Inhibited	-2.261	1.4E-23	22.8539
IL13	cytokine	Inhibited	-2.232	5.58E-22	21.2534
PDGF BB	complex	Inhibited	-2.554	9.17E-22	21.0376
CTNNB1	transcription regulator	Inhibited	-3.226	9.69E-22	21.0137
JUN	transcription regulator	Inhibited	-2.949	3.97E-21	20.4012
TWIST1	transcription regulator	Inhibited	-3.279	6.6E-21	20.1805
FGF2	growth factor	Inhibited	-2.895	8.4E-19	18.0757
NFkB (complex)	complex	Inhibited	-2.366	1.05E-18	17.9788
Ins1	other	Inhibited	-2.019	1.93E-18	17.7144
CD44	other	Inhibited	-4.719	1.28E-17	16.8928
EGFR	kinase	Inhibited	-2.276	2.88E-17	16.5406

IKBKB	kinase	Inhibited	-2.605	9.04E-17	16.0438
SMAD3/4	transcription regulator	Inhibited	-4.377	1.32E-15	14.8794
SREBF1	transcription regulator	Inhibited	-2.627	1.09E-14	13.9626
IL1	group	Inhibited	-4.055	2.07E-14	13.6840
Vegf	group	Inhibited	-3.767	7.78E-14	13.1090
HIF1A	transcription regulator	Inhibited	-2.456	2.55E-13	12.5935
FOXO1	transcription regulator	Inhibited	-2.344	3.22E-13	12.4921
Jnk	group	Inhibited	-3.966	4.13E-13	12.3840
Brd4	kinase	Inhibited	-3.59	7.15E-13	12.1457
ERK	group	Inhibited	-3.653	6.64E-12	11.1778
MTPN	transcription regulator	Inhibited	-4.216	7.01E-12	11.1543
Akt	group	Inhibited	-3.864	1.28E-11	10.8928
P38 MAPK	group	Inhibited	-3.918	2.97E-11	10.5272
ROCK1	kinase	Inhibited	-3.334	8.05E-11	10.0942
PI3K (complex)	complex	Inhibited	-3.78	8.88E-10	9.0516
LDL	complex	Inhibited	-2.146	1.51E-09	8.8210
FOXM1	transcription regulator	Inhibited	-3.689	1.76E-09	8.7545
CCN2	growth factor	Inhibited	-2.934	5.54E-09	8.2565
PRKCD	kinase	Inhibited	-3.004	1.63E-08	7.7878
SYVN1	transporter	Inhibited	-2.73	1.37E-07	6.8633
Collagen type I	complex	Inhibited	-2.094	4.65E-07	6.3325
HRG	other	Inhibited	-2.538	6.81E-07	6.1669
mir-223	microRNA	Inhibited	-4.121	9.68E-07	6.0141
Ap1	complex	Inhibited	-3.102	0.00000198	5.7033
CREBBP	transcription regulator	Inhibited	-2.094	0.00000328	5.4841
HSF1	transcription regulator	Inhibited	-2.162	0.00000447	5.3497
RAS	group	Inhibited	-2.933	0.0000112	4.9508
CYP2E1	enzyme	Inhibited	-2.607	0.000104	3.9830
